# Polymyxin Acute Kidney Injury: Dosing and Other Strategies to Reduce Toxicity

**DOI:** 10.3390/antibiotics8010024

**Published:** 2019-03-14

**Authors:** Roger L. Nation, Maria Helena P. Rigatto, Diego R. Falci, Alexandre P. Zavascki

**Affiliations:** 1 Drug Delivery, Disposition and Dynamics, Monash Institute of Pharmaceutical Sciences, Monash University, Parkville 3052, Australia; roger.nation@monash.edu; 2 Infectious Diseases Service, Pontifícia Universidade Católica do Rio Grande do Sul, Porto Alegre 90035-903, Brazil; mhprigatto@gmail.com; 3 Infectious Diseases Service, Hospital de Clinicas de Porto Alegre, Porto Alegre 90035-903, Brazil; diego.falci@gmail.com; 4 Health and Human Development Post-Graduation Program, Universidade La Salle, Canoas 92010-000, Brazil; 5 Department of Internal Medicine, Universidade Federal do Rio Grande do Sul, Porto Alegre 90035-903, Brazil

**Keywords:** colistin, polymyxin B, acute kidney injury, dosing, pharmacokinetics/pharmacodynamics

## Abstract

Polymyxins are valuable antimicrobials for the management of multidrug-resistant Gram-negative bacteria; however, nephrotoxicity associated with these drugs is a very common side effect that occurs during treatment. This article briefly reviews nephrotoxic mechanisms and risk factors for polymyxin-associated acute kidney injury (AKI) and discusses dosing strategies that may mitigate kidney damage without compromising antimicrobial activity. Polymyxins have a very narrow therapeutic window and patients requiring treatment with these drugs are frequently severely ill and have multiple comorbidities, which increases the risk of AKI. Notably, there is a significant overlap between therapeutic and toxic plasma polymyxin concentrations that substantially complicates dose selection. Recent dosing protocols for both colistin and polymyxin B have been developed and may help fine tune dose adjustment of these antibiotics. Minimizing exposure to modifiable risk factors, such as other nephrotoxic agents, is strongly recommended. The dose should be carefully selected, particularly in high-risk patients. The administration of oxidative stress-reducing drugs is a promising strategy to ameliorate polymyxin-associated AKI, but still requires support from clinical studies.

## 1. Introduction

Polymyxin antibiotics were introduced in the 1950s for the treatment of Gram-negative infections. In the 1970s, they were progressively abandoned as a therapeutic due to the development and launch of newer and less toxic antibiotics [1,2]. Owing to the emergence of carbapenem-resistant Gram-negative bacteria near the end of the last century, notably in the early 2000s, and the lack of new therapeutic options, polymyxins re-emerged in clinical practice [1,2]. Since then, the toxicity associated with these compounds, particularly nephrotoxicity, has been of great concern.

As reported in earlier studies, the emerging experience with both polymyxins, colistin (administered as the prodrug colistimethate [CMS]) and polymyxin B, indicated that up to half of the patients receiving these drugs presented nephrotoxicity [3]. However, it became clear that multiple definitions for nephrotoxicity impaired a more accurate estimate of the real incidence of acute kidney injury (AKI) in patients treated with polymyxins [3]. More recent studies using standardized criteria for AKI, such as Kidney Disease: Improving Global Outcomes (KDIGO), Acute Kidney Injury Network (AKIN) and Risk, Injury, Failure, Loss of kidney function, and End-stage kidney disease (RIFLE), have been published, among which, a recent meta-analysis showed that the occurrence of AKI remained undesirably high with mean rates of 31.3%, 32.6% and 39.4%, respectively [4]. Patients treated with colistimethate may have higher AKI rates than those treated with polymyxin B [5], although a more recent meta-analysis showed that there was no significant difference of nephrotoxicity between polymyxins [4].

Strategies to avoid or minimize nephrotoxicity have become a challenge for clinicians treating patients with polymyxins since many associated risk factors for AKI are very common in the typical patient who needs a polymyxin-based therapy, and most of them are non-modifiable. The purpose of this article is to discuss the dosing management of these valuable antibiotics in order to avert or decrease kidney injury in patients treated with polymyxins. Other strategies to decrease nephrotoxicity and a brief review of the mechanisms of toxicity and risk factors for polymyxin-associated AKI are also presented.

## 2. Mechanisms of Nephrotoxicity

Knowledge of the polymyxin-induced mechanisms of nephrotoxicity has significantly increased in the last decade, helping in the exploration of different strategies to reduce renal damage caused by these drugs. A starting point in the pathway of events that leads to nephrotoxicity is avid proximal tubule reabsorption that both polymyxins (polymyxin B and colistin) undergo in the kidneys [6]. In this process, there is a substantial intracellular accumulation of the drug which is mediated by the endocytic receptor, megalin, as well as other transporters [7]. The high intracellular concentration of polymyxins has been demonstrated in both human (HK-2) and rat (NRK-52E) kidney cells, which allowed experiments in animal and cell culture models to further explore this phenomenon [8,9]. The accumulation of polymyxins leads to drug-induced cell apoptosis which in turn results in decreased renal function and histopathological damage. Animal studies have shown that dilated renal tubules, cast formation and tubular necrosis occur in a dose- and time-dependent fashion after exposure to polymyxins [10]. The major pathways that result in kidney cell apoptosis involve the activation of death receptors, mitochondrial damage, and changes in endoplasmic reticulum function and the autophagy process [8,10,11].

The death receptor pathway is one of the most sensitive in the cell damage process [10]. Increased membrane-anchored FasL in rats exposed to polymyxins binds to Fas (death receptor), then triggers caspase-8 activation and, subsequently, caspase-3 activation, which is the final executioner of DNA fragmentation and cellular apoptosis [8]. The activation of the death receptor pathway liberates reactive oxygen species, increasing oxidative stress, which in turn contributes to mitochondrial fragmentation.

Mitochondrial morphology is significantly changed when exposed to polymyxins, changing from its normal filamentous form to a fragmented version. The change in mitochondrial morphology results in the loss of its membrane potential and increased superoxide production, which leads to more mitochondrial damage. This intrinsic mitochondrial pathway activates proapoptotic proteins, such as caspase-9, which also culminates in caspase-3 activation and cell death [8]. Moreover, the endoplasmic reticulum, when under prolonged stress, activates proapoptotic genes, such as growth arrest and DNA damage 153 (GADD153), which activates caspase-12, followed by the activation of downstream caspases, leading to cellular apoptosis [10]. Finally, the normal autophagy process, responsible for removing damaged proteins and organelles, can be decreased when exposed to high doses of polymyxins. This contributes to a malfunctioning of the balance between apoptosis and autophagy and leads to worse control of cell damage [10].

In summary, polymyxins induce pan-caspase activation in a time- and concentration-dependent manner through different pathways, which ultimately leads to cell apoptosis. Oxidative stress generated by this process is responsible for further activating these pathways, maintaining the cycle that leads to kidney cell death [8]. Ultimately, understanding the mechanisms of polymyxin renal toxicity should help point to future strategies in the management of patients receiving these drugs.

## 3. Risk Factors for Nephrotoxicity

The development of AKI is the most common and undesired adverse effect of polymyxins. The occurrence of AKI during treatment is associated with worse prognosis, including higher mortality rates [12,13,14]. Furthermore, among patients who developed AKI during polymyxin therapy, a higher incidence of chronic renal failure has been recently demonstrated [15,16]. Thus, decreasing the incidence of AKI during treatment with polymyxins may have short- and long-term clinical impacts in patients treated with these antibiotics.

In order to limit kidney injury during treatment with polymyxins, lowering exposure to other risk factors for AKI is an appealing strategy. Unfortunately, many polymyxin-associated AKI risk factors reported are not modifiable. In addition, although some chronic comorbid conditions, notably diabetes, have been identified as risk factors for AKI [17,18,19,20,21], patients requiring treatment with polymyxins are commonly severely ill and have multiple chronic conditions that together may increase drug-induced injury to the kidneys [18,22,23,24,25,26,27].

Among the non-modifiable risk factors, higher age has been reported in many studies [14,17,19,22,28,29,30]. However, it is not known whether there is a linear effect of age on the risk of AKI or whether the risk may increase over a given age. It is interesting that higher weight also increases the risk of AKI and this effect has been shown to be independent of the dose administered to the patients [14,19,31]. In addition, hypoalbuminemia has been shown to increase the risk of AKI in some studies [20,25,32,33], although the mechanism for the association is not known [33]. At this time, it is not considered appropriate to modify the daily dose of colistimethate in patients with hypoalbuminemia [34].

There are a few potentially modifiable risk factors that have been identified. One potentially modifiable risk factor that has been reported is the use of concomitant nephrotoxic drugs. Concomitant use of one or more non-specified nephrotoxic drug has been reported in some studies [17,23,27,35], while loop diuretics [18,36], calcineurin inhibitors [20], non-steroidal anti-inflammatory drugs [32] and intravenous contrast media [37] have been identified as risk factors among non-antimicrobial drugs. Vancomycin has been reported in some studies [31,37,38,39], while aminoglycosides [30] and rifampin [35] have been reported in single studies, although randomized clinical trials with rifampin in association with colistin have not demonstrated a higher incidence of nephrotoxicity in patients in rifampin arms [40,41]. In fact, there is some incipient evidence that combination therapy may decrease the risk of AKI. In a recent randomized clinical trial comparing colistin with colistin plus meropenem for severe Gram-negative infections, the incidence of AKI was significantly higher in the monotherapy group (48%) compared with the combination group (29%) (*p* = 0.001), which was determined by differences in Injury (14% vs. 6%, respectively) and Failure (17% vs. 8%, respectively), categories of RIFLE [42]. There were no differences between groups regarding colistin dose (although total cumulative doses were only reported for patients alive on day 14) and concomitant use of nephrotoxic drugs [42]. More recently, a meta-analysis evaluating the safety and efficacy of colistin alone or in combination in adults with *Acinetobacter baumannii* infection has also found that the incidence of nephrotoxicity was higher in the monotherapy group (odds ratio = 1.66, 95% confidence interval = 0.99–2.78, *p* = 0.05) [43]. Additionally, although it may be associated with the oxidative stress-reducing properties of minocycline [44], a combination of colistin with minocycline was shown to reduce the incidence of AKI in an observational study [45]. We speculate that combination therapy, regardless of its impact on overall mortality, owing to higher microbiological effectivity, might result in less general inflammatory response and/or earlier recovery from sepsis, thus decreasing the dysfunction of organs, including the kidneys.

The most important variable associated with the development of nephrotoxicity is polymyxin dose [14,20,24,35,36,37,38,39,46,47]. Although evaluated in a few studies, the administration of loading doses has been found to increase the risk of nephrotoxicity in some studies [28,48], but not others [18,23,38,49]. Additionally, and possibly related to total drug exposure, a longer duration of treatment has been identified by some authors [21,29]. The issue of doses and plasma polymyxin concentrations [27,50] will be discussed in the following section.

## 4. Dosing Strategies to Reduce Nephrotoxicity

While a greater amount of preclinical, clinical pharmacological and toxicological data are available for colistin than for polymyxin B, it is evident that both agents have very low therapeutic indices. For each agent, the substantial overlap in the plasma concentrations that are generally regarded as being required for the desired antibacterial effect and those that may predispose to nephrotoxicity mandates that dosing strategies must be carefully considered, as highlighted in the recently published international consensus guidelines for the optimal use of the polymyxins [51]. In regard to careful selection of a dosing strategy for each patient, it is important to be aware that sepsis arising from an inadequately treated Gram-negative infection can also lead to kidney injury [52]. At the outset, it is both relevant and important to consider the data that indicate the narrowness of the therapeutic window of each of the polymyxins.

### 4.1. Colistin and Polymyxin B: Drugs with Very Narrow Therapeutic Windows

**Figure 1:**
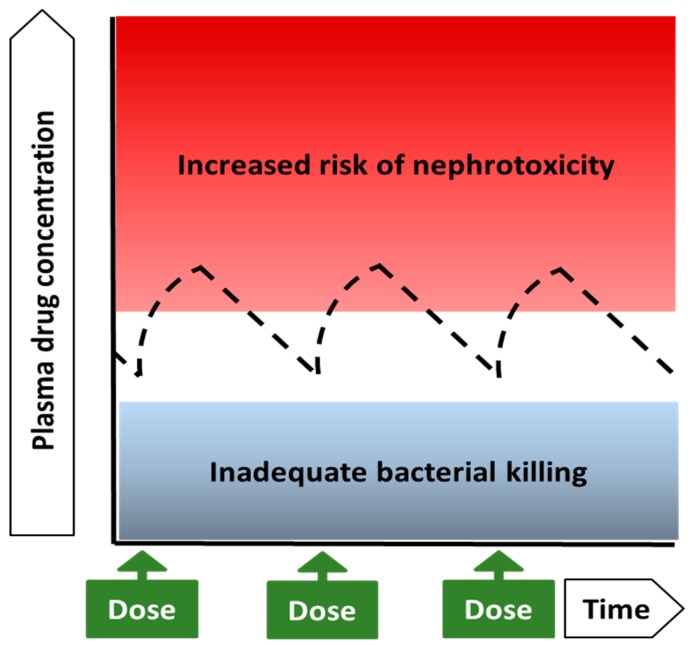
Diagrammatic representation of the very narrow therapeutic window of colistin, which is based on two key considerations. The first consideration was the translation of PK–PD data from murine thigh infection studies, in which subcutaneous colistin was used against infections caused by either *P. aeruginosa* or *A. baumannii* [57]. In this infection model, an average value of approximately 12 for the ratio of the area under the free (unbound) plasma concentration versus the time curve over 24 h (*f*AUC_24_) to the minimum inhibitory concentration (MIC) of the pathogen (*f*AUC_24_/MIC) was required for a 2-log bacterial kill each of *P. aeruginosa* and *A. baumannii* [57]. Given that colistin is approximately 50% bound in plasma of critically ill patients, a *f*AUC_24_/MIC of 12 determined in mice translates to a AUC_24_/MIC of 24 when considering the total plasma concentration in human patients [53,54]. Since an AUC_24_ of 24 mg·h/L corresponds to an average steady-state plasma concentration (C_ss,avg_) of 1 mg/L (i.e., 24 mg·h/L divided by 24 h), translation of the murine PK–PD data to critically ill patients suggests that, from an antibacterial perspective, the target C_ss,avg_ should correspond to the MIC of the infecting organism. Thus, based on PK–PD data from thigh infection models in mice, a C_ss,avg_ of 2 mg/L may be considered appropriate for treatment of an infection caused by an organism with an MIC of 2 mg/L, which is the clinical breakpoint for *P. aeruginosa* and *A. baumannii* and the epidemiological cut-off value for *K. pneumoniae* and several other Gram-negative pathogens [56]. The second important consideration in proposing an initial target plasma colistin C_ss,avg_ of 2 mg/L was the clinical PK–TD data on the relationship between the plasma colistin concentration and risk of nephrotoxicity in critically ill patients. These data indicate that nephrotoxicity can occur even in the face of relatively low plasma colistin concentrations and that the likelihood of nephrotoxicity substantially increases above a plasma colistin C_ss,avg_ of approximately 2–3 mg/L [27,50,58].

For colistin, an average steady-state plasma concentration (C_ss,avg_) of 2 mg/L has been suggested as a target level of exposure at the initiation of therapy, a time when the minimum inhibitory concentration (MIC) of the organism causing infection may not be known [53,54]. Even if an estimate of MIC is available at that time, it must be recognized that MIC measurements are subject to error [55]. The proposed plasma colistin C_ss,avg_ target of 2 mg/L corresponds to the MIC clinical breakpoint for *Pseudomonas aeruginosa* and *A. baumannii* and the epidemiological cut-off value for *Klebsiella pneumoniae* and several other Gram-negative pathogens [56]. The target to guide therapy at the initiation of treatment was based on pharmacokinetic–pharmacodynamic (PK–PD) data from murine models of infection with *P. aeruginosa* and *A. baumannii* [57] and pharmacokinetic–toxicodynamic (PK–TD) data on the relationship between the plasma colistin concentration and risk of nephrotoxicity in critically ill patients [27,50,58] that together indicate the very narrow therapeutic window for colistin, as shown diagrammatically in Figure 1.

A tentative target therapeutic range for plasma polymyxin B AUC_24_ at a steady state of 50–100 mg·h/L has recently been proposed; this corresponds to a plasma polymyxin B C_ss,avg_ range of approximately 2–4 mg/L [59]. The lower end of this range was based on the aforementioned murine PK–PD data for colistin against *P. aeruginosa* and *A. baumannii* [57], assuming similar in vivo antibacterial effects of colistin and polymyxin B, and PK–PD data for polymyxin B against *K. pneumoniae* infection in thighs of mice [60]. The upper end of the proposed polymyxin B concentration range was from a PK–TD meta-analysis of previously published articles reporting nephrotoxicity rates in patients treated with the drug. The meta-analysis revealed a statistically significant linear relationship between the percentage of patients with a ≥25% decrease in creatinine clearance during polymyxin B therapy and the plasma polymyxin B C_ss,avg_ [59]. The C_ss,avg_ associated with rates of mild nephrotoxicity (≤25% decrease in creatinine clearance) in ≤40% of patients was estimated to be ~4 mg/L; however, as discussed above for colistin nephrotoxicity, mild nephrotoxicity was observed in some patients with lower concentrations.

Clearly, based on PK–PD data from murine thigh infection models and clinical PK–TD analyses, plasma concentrations associated with nephrotoxicity overlap with those for antibacterial effect. It is important to note that experimental lung infections in mice were substantially less responsive than thigh infections to the systemic administration of each of the polymyxins [57,60]. This implies that the ability of the parenteral administration of polymyxin to achieve concentrations in plasma associated with antibacterial effects in the lung, while having acceptable potential for nephrotoxicity, is severely compromised. In other words, the murine lung infection studies suggest greater overlap than that based on the murine thigh infection studies in the plasma concentrations associated with the desired (i.e., antibacterial) effect and the major undesired (i.e., nephrotoxic) effect.

### 4.2. Implications of the Narrow Therapeutic Range for Dosing of Polymyxins

#### 4.2.1. Choice of Polymyxin

As discussed above, there may be a greater margin of safety for polymyxin B compared to colistin. Studies reported over the last decade and meta-analyses on the derived data suggest that the risk of nephrotoxicity may be lower with polymyxin B [5,17,18,29,39,48,61,62], although a recent meta-analysis that included a large number of older studies suggested that there was no difference in nephrotoxicity prevalence between the two polymyxins [4]. It should be noted that several of the studies, especially from earlier literature, are difficult to interpret due to confounding influences, diversity in definitions of nephrotoxicity and lack of detail [63]. In addition, as reviewed previously [64,65] and discussed below, polymyxin B has PK characteristics that render it generally easier to use than is the case for colistin. Currently, it is suggested that where clinicians have a choice between the two polymyxins, polymyxin B is the preferred agent for invasive infections [51,65]. Colistimethate (the inactive prodrug of colistin [66,67]) is preferred for urinary tract infections, as the extensive urinary excretion of the prodrug followed by spontaneous conversion to colistin within the urinary tract leads to high concentrations of colistin in urine [68,69]. Colistimethate may also be the preferred polymyxin preparation for administration via the intraventricular or intrathecal route [70,71,72,73,74,75,76]; there is less published information relating to the administration of polymyxin B via these routes.

#### 4.2.2. To Load or Not to Load?

As discussed in Section 3, the initiation of polymyxin therapy with a loading dose has not been conclusively shown to be a risk factor for nephrotoxicity. Due to the time required for plasma colistin concentrations to rise after initiating colistimethate therapy, a loading dose should be used [51]. Even if this increased the risk of nephrotoxicity, the harm of a delay in achieving therapeutic plasma levels may be worse. For polymyxin B, in the absence of evidence of increased risk for nephrotoxicity, a loading dose is recommended, especially for severe infections and for bacteria with a high MIC.

#### 4.2.3. Selection of the Daily Maintenance Dose at Initiation of Therapy and Ongoing Optimization

##### Colistin

Colistin displays rather complex PK characteristics [1,53,54,64,77,78]. Following the intravenous administration of colistimethate to patients with good renal function, extensive renal excretion of the prodrug occurs and only a relatively small proportion (20–30% or less) of the administered dose of colistimethate is available for conversion to colistin within the body (excluding the urinary tract). Renal function, as assessed by creatinine clearance, is the only patient factor that has been shown to influence the plasma colistin C_ss,avg_ during ongoing therapy with colistimethate [54,79]. As clinical PK–TD analyses have shown the direct relationship between the plasma colistin C_ss,avg_ and the risk of nephrotoxicity [27,50,58], at the initiation of therapy, it is important to select a daily maintenance dose of colistimethate that is considered appropriate given the baseline creatinine clearance of the patient [51]. Based on a population PK analysis of data from 214 adult critically ill patients, an algorithm incorporating creatinine clearance has recently been developed to enable the selection of a daily dose of colistimethate to achieve a desired plasma colistin C_ss,avg_ (2 mg/L, or a lower concentration if that is deemed appropriate) [54]. The algorithm was developed such that, across various categories of creatinine clearance values, >80% of patients would achieve the desired plasma colistin C_ss,avg_ and, to minimize the potential for colistin-associated nephrotoxicity, <30% of patients would achieve a plasma exposure of greater than twice the desired target.

It is essential to appreciate the following points regarding the application of the algorithm [54]. First, because of the very wide inter-patient variability in the apparent clearance of formed colistin (even at a given creatinine clearance) and the narrow therapeutic window of colistin, it is not possible that less than 30% of patients are likely to achieve greater than twice the desired antibacterial target while simultaneously at least 80% are likely to achieve the desired antibacterial target. Second, because of the rapid clearance of colistimethate in patients with creatinine clearance, ≥80 mL/min, it is only possible to achieve a plasma colistin C_ss,avg_ of 2 mg/L in less than 40% of such patients, even with a daily dose of 360 mg of colistin base activity (corresponding to ~10.9 million international units (IU) per day). It is regarded as unwise to increase the daily dose above this level as that may increase the risk of nephrotoxicity. Third, it is axiomatic that the application of the algorithm provides no assurance that a plasma colistin C_ss,avg_ equal to or greater than the desired target will be achieved or indeed that a plasma concentration more than twice the target will not be achieved. Finally, there is no guarantee that the achievement of a plasma colistin C_ss,avg_ less than twice the desired target, or indeed less than the target itself, will not predispose to nephrotoxicity.

The algorithm is simply a tool to enable the estimation of what may be regarded as a reasonable daily maintenance dose at the initiation of therapy [54]. The algorithm may also be used for renal-based dose adjustments during ongoing therapy if there is a change in creatinine clearance [51]. For example, in the event of a decrease in creatinine clearance (for whatever reason) where a decision is made to continue therapy with colistimethate, the daily dose should generally be adjusted downwards to maintain the original target concentration. If a reliable estimate of the MIC of the infecting organism and/or the clinical circumstances suggest that a lower plasma concentration may be adequate, it may be appropriate to apply the algorithm to target a plasma colistin C_ss,avg_ lower than the original target concentration. These approaches are intended to minimize the potential for a colistin-associated exacerbation of the already established renal function decline.

As discussed above, the very substantial inter-patient variability in the apparent clearance of colistin (and, hence, the plasma colistin C_ss,avg_ achieved from a particular daily dose of colistimethate) and the narrow therapeutic window impose limitations on the application of the algorithm [54]. It is highly desirable that ongoing therapy with colistimethate is guided by reliable measurement of the plasma colistin concentration via timely access to a therapeutic drug monitoring/management (TDM) service [51], as available in some settings [80,81,82,83]. Knowledge of the plasma colistin concentration enables the optimization of the daily dose of colistimethate to maximize the likelihood of a desired antibacterial outcome, while minimizing the risk of nephrotoxicity. Unfortunately, reliable determination of the plasma colistin concentration in samples collected from patients receiving colistimethate is extremely challenging, not least because the circulating concentration of colistimethate is higher than that of colistin, especially during the first several hours of a dosage interval. Microbiological assays cannot be used because of the ongoing conversion of colistimethate to colistin during the incubation procedure of the assay [67] and issues related to the sensitivity and specificity of such assays. Even with chromatographic assay methods, there is a need for very careful sample collection, processing, storage and analysis procedures to avoid ongoing conversion of the prodrug to colistin after a blood sample has been collected from the patient [64,84,85,86]. Such difficulties do not arise with polymyxin B because it is not administered as a prodrug; this is one of several features that favors the use of polymyxin B over colistimethate for most types of invasive infections [64].

##### Polymyxin B

Typically, less than 5% of an intravenous dose of polymyxin B is excreted in urine in the form of the unchanged drug [87,88]. It is, therefore, not surprising that renal function, as assessed by creatinine clearance, does not influence the clearance of polymyxin B to a clinically significant degree [87,88,89,90,91,92]. At the initiation of maintenance therapy, a daily dose of 1.5 to 3 mg/kg (equivalent to 15,000 to 30,000 IU/kg) of polymyxin B is generally used, depending on the MIC (often not known at initiation of therapy), and the site and severity of infection. Renal-based dose selection is not relevant for polymyxin B because of the lack of clinically significant influence of creatinine clearance on the clearance of the drug [51]. There is limited experience with absolute doses ≥200 mg per day and infusion-related adverse effects may increase with such doses [23,93]. If a decrease in creatinine clearance occurs during therapy, the polymyxin B daily dose should not be decreased, particularly in a patient with a life-threatening or deep-seated infection or if the pathogen’s MIC is >1 mg/L.

Recently, Lakota et al. [59] investigated the application of adaptive feedback control (AFC) in silico to optimize and individualize dosage regimens of polymyxin B, with the aim of maximizing the potential antibacterial effect and minimizing the likelihood of nephrotoxicity. AFC, an approach involving the use of plasma drug concentration measurements (i.e., PK samples) to refine and feedback estimates of individual PK characteristics to the AFC algorithm, is generally a substantially more informative dose optimization approach than traditional TDM [94]. In view of the less complex PK and assay issues for polymyxin B relative to colistimethate/colistin, polymyxin B is likely the superior of the two agents for the application of AFC.

In their analysis, Lakota and co-workers [59] used a previously developed population PK model [88] to develop an AFC algorithm for polymyxin B which was evaluated using Monte Carlo simulations of 5000 simulated patients. Without AFC, a daily polymyxin B dose of 2 mg/kg, computed based on the population average polymyxin B clearance of 0.0276 L/h/kg to achieve a plasma polymyxin B C_ss,avg_ of 3 mg/L (i.e., the middle of the target window of 2–4 mg/L discussed in Section 4.1) was applied to all simulated patients. This ‘one dose fits all’ daily dose resulted in only 71% of simulated patients achieving a plasma polymyxin B C_ss,avg_ within the 2–4 mg/L range. Worryingly, 19.8% of simulated patients had a C_ss,avg_ above 4 mg/L and the highest predicted C_ss,avg_ was >8 mg/L. When individualized doses were computed using the AFC algorithm with only one PK sample collected at 12 h after the first dose in the regimen, 93.6% of simulated patients achieved exposures within the target C_ss,avg_ window of 2–4 mg/L and only 5.0% of patients had exposures above 4 mg/L. The corresponding probability of target attainment with a single PK sample collected 24 h after the first dose was 95.3% with 2.5% above the upper end of the target window. With 3 or 4 PK samples collected during the first day, the probability of target window attainment increased to >99% [59]. Clearly, this approach has substantial promise to aid the selection of optimized and individualized polymyxin B daily doses. Prospective clinical studies to evaluate AFC for polymyxin B therapy are eagerly awaited.

#### 4.2.4. Dosage Interval and Infusion Duration

Traditionally, dosing guidelines have proposed that the intravenous administration of the chosen daily dose of a polymyxin should be in 2 or 3 divided doses, i.e., a dosage interval of 12 or 8 h. Based on the established clinical practice of the once-daily administration of aminoglycoside antibiotics in order to decrease the potential for nephrotoxicity [95,96], there has been interest in the possibility that the influence of dosage interval may also apply to polymyxin-induced nephrotoxicity. Wallace et al. [97] were the first to explore this possibility for colistimethate in a rat model. In that study, the regimen corresponding to once-daily administration in humans resulted in a greater number and severity of renal lesions than in rats that received the same daily dose in a regimen corresponding to twice-daily administration in humans. It was proposed that extended-interval dosing resulted in higher concentrations of colistimethate and colistin in renal tubular cells, which predispose to nephrotoxicity due to concentration-dependent toxicity [97]. In contrast, Abdelraouf et al. [98], who also employed a rat model, found lower nephrotoxicity of polymyxin B when administered in a once-daily regimen relative to the same daily dose administered in divided doses, 6 hourly. It was suggested that this finding may have been the result of a greater saturation of the carrier-mediated uptake of polymyxin B from tubular urine into cells with the once-daily regimen [98], but other mechanisms may have been involved [63]. Importantly, in a multicenter retrospective clinical study, Okoduwa et al. [99] observed, using propensity score matching of 100 patients in each regimen, that nephrotoxicity was more common with once-daily than twice-daily dosing of polymyxin B (47% versus 17%, respectively; *p* = 0.0005). This finding is consistent with the results of Wallace et al. who used a rat model [97]. Until the results of appropriately powered prospective clinical studies are available, it is prudent to divide the daily dose to minimize the risk of nephrotoxicity, an approach which may also reduce the potential for the development of bacterial resistance based on the results of in vitro studies against *P. aeruginosa* [100,101].

Due to the concentration-dependent toxic effect of polymyxin on renal tubular cells [11,98], the likelihood of nephrotoxicity may possibly increase as the infusion duration is decreased, i.e., a shorter duration of infusion leads to a higher plasma concentration during and soon after the infusion. Unfortunately, the possible effect of the duration of infusion of each maintenance dose on the risk of nephrotoxicity has not been evaluated in appropriately designed and powered clinical studies. It is recommended that colistimethate be infused over 0.5 to 1 h, and polymyxin B over at least 1 h [51].

## 5. Other Strategies to Reduce Nephrotoxicity

Obviously, general measures such as strict monitoring of renal function during therapy as well as maintaining an adequate fluid and electrolyte balance are essential components of the strategies that may reduce the risk of polymyxin-associated AKI [63,102]. As discussed in Section 3, there are a few potentially modifiable risk factors for polymyxin-associated AKI, such as avoiding the co-administration of other known nephrotoxic agents whenever possible, and these should be addressed [51]. Also discussed in that earlier section is the emerging evidence that combination therapy with colistin may be associated with a lower potential for nephrotoxicity relative to colistin monotherapy, although the clinical studies were largely retrospective in nature and/or nephrotoxicity was not the primary or even secondary outcome measure. The potential benefit of combination therapy in terms of toxicological outcomes requires further evidence from properly designed studies.

Interventions tested to attenuate polymyxin nephrotoxicity have mainly focused on reducing oxidative stress mechanisms enhanced by these antibiotics. Many anti-oxidative stress agents have been tested so far, mostly on animal models. The main results are summarized in Table 1. Overall, the concomitant use of these agents with polymyxins has led to the reduced production of inflammatory and oxidative stress biomarkers [103,104,105,106]. Attenuation of both cellular apoptosis and histopathological changes in mice kidneys has been shown as well [103,105,107,108,109]. Nevertheless, not all studies have shown significant changes in serum creatinine values when comparing polymyxins prescribed alone or combined with anti-oxidant drugs [106,110], possibly because after a renal insult, there is a delay in the response of the serum creatinine concentration.

Clinical studies exploring this issue are still scarce. Ascorbic acid has been the most explored agent due to its safety profile and encouraging results when used with other nephrotoxic agents. It is a chain-breaking antioxidant that has previously been shown to reduce contrast medium-associated nephrotoxicity [111]. One prospective cohort study of septic patients treated with colistin showed that ascorbic acid was an independently protective factor for AKI [112]. However, a randomized clinical trial (RCT) that enrolled 28 patients testing the addition of 2 g of ascorbic acid (the same dose that was demonstrated to be effective in preventing contrast nephrotoxicity) to colistin every 12 h did not show any benefits in renal function or a reduction of urinary biomarkers for nephrotoxicity in the intervention group [113]. Although the small number of participants may have limited the power of the study, the absolute difference in reducing the nephrotoxicity incidence was only 6.2%, favoring the group that received ascorbic acid. The clinical significance of this small effect could be questionable.

Melatonin is another well-known agent for its anti-oxidative stress properties. Its renal protective effect has been explored in humans and animal models on a variety of conditions such as diabetes, hypertension and drug-induced nephropathy [117]. In one study, it showed a protective effect against colistin-induced AKI in rats [109]. There is currently one RCT registered in ClinicalTrials.gov (NCT03725267) testing the potential effect of melatonin versus placebo in polymyxin-induced nephrotoxicity.

Nephrotoxicity is one of the most important drawbacks of polymyxin use and the development of strategies to overcome this issue is urgently needed. However, most studies are still in the pre-clinical phase and there is currently insufficient evidence to support the routine implementation of any of these strategies. This is a vast clinical research field yet to be explored.

## 6. Conclusions

Despite the recent launch of newer antimicrobial drugs with activity against some carbapenem-resistant Gram-negative bacteria, polymyxins still have a major role in the therapeutic arsenal against these organisms and the attenuation of nephrotoxicity of these compounds will continue to challenge clinicians. The optimization of antibacterial activity without increasing AKI risk is a very difficult task at the bedside due to the narrow therapeutic window of polymyxins. Ultimately, clinicians will always be confronted with the dilemma of augmenting drug exposure to improve the odds of microbiological and, hopefully, clinical success, and, at the same time, increase the probability of AKI, which is associated with worse outcomes. Minimizing exposure to modifiable risk factors, such as concomitant nephrotoxic agents, including antibiotics such as vancomycin, along with general measures such as strict monitoring of renal function and the maintenance of an appropriate fluid and electrolyte balance during therapy are important to reduce the risk of AKI. The use of oxidative stress-reducing drugs is a promising therapy but still requires more evidence from clinical studies. Optimizing therapy with combination regimens to decrease overall AKI rates also deserves further attention. Finally, dose selection should be rigorously defined in order to avoid the excessive risk of AKI without compromising antibacterial activity, particularly in high-risk patients such as the elderly, obese, and those with multiple comorbidities and requiring other nephrotoxic drugs.

## Figures and Tables

**Table 1:** Summary of the studies evaluating interventions for the prevention of polymyxin nephrotoxicity.

Study	Intervention/Exposure Factor	Main Results
**Animal Models**		
Li et al., 2019 [114]	Cytochrome C	- Cytochrome C (a megalin ligand) decreased the accumulation of polymyxin B in the kidney and 24-h *N*-acetyl-β-d-glucosaminidase (NAG) in a dose-dependent manner. - Histological damage was reduced. - No significant differences in serum creatinine, blood urea nitrogen (BUN), and blood β2- microglobulin were seen in the groups that received Cytochrome C compared to the one that received polymyxin B alone.
Ceylan et al., 2018 [108]	*N*-acetylcysteine (NAC)	- Colistin increased the apoptosis index and renal histological damage score significantly and these changes were reduced with NAC co-treatment. - There was no difference between groups regarding total antioxidant and total oxidant status in the kidneys.
Dai et al., 2017 [105]	Baicalein	- Baicalein attenuated colistin-induced oxidative and nitrative stress, apoptosis, the infiltration of inflammatory cells, and caused decreases in interleukine-1β and tumor necrosis factor-α levels (all *p* < 0.05 or 0.01) in kidney tissues. - Baicalein attenuated colistin-induced kidney tissue damage on histopathological analysis, in a dose-dependent manner.
Azad et al., 2017 [103]	Methionine	- Histological: polymyxin-induced nephrotoxicity in mice was ameliorated by methionine in a dose-dependent manner. - Attenuation of polymyxin-induced mitochondrial superoxide production in rat kidney cells was observed following pretreatment with methionine. - Pharmacokinetics of polymyxin B in rats were not affected by methionine.
Hassan et al., 2017 [110]	Silybin	- Colistin-alone group showed an increase in NAG (*p* < 0.01) and reduction of renal function compared to other groups (control, vehicle and colistin plus silybin) (*p* < 0.001), but no difference was found in a direct comparison of colistin plus sybilin group with the colistin-alone group.
Arslan et al., 2016 [107]	Luteolin	- Colistin-treated group had statistically higher number of apoptotic cells compared to the other three groups (luteolin, luteolin plus colistin and control) (*p* = 0.0001) and was the only group to increase serum creatinine values compared to pre-treatment levels. - Renal histological damage was also measured and the score of the colistin-treated group was higher as compared to other groups.
Dai et al., 2015 [104]	Lycopene	- Biomarkers of oxidative stress and apoptosis were attenuated in the kidneys of colistin-treated mice by the co-administration of lycopene (5 or 20 mg/kg).
Ozkan et al., 2013 [115]	Grape seed proanthocyanidin extract (GSPE)	- Colistin + GSPE group showed significant decreases in BUN levels; creatinine levels; renal histopathological scores; and terminal deoxynucleotidyltransferase-mediated dUTP-biotin nick end labeling, caspase 1 and 3, calpain 1, iNOS, and eNOS staining when compared to the colistin group alone.
Yousef et al., 2012 [116]	Ascorbic acid	- 24-h urinary excretion of NAG was significantly lower in the groups that received ascorbic acid compared to colistin alone (*p* < 0.01). - The percentage of apoptotic cells decreased in the ascorbic acid group in a dose-dependent manner (*p* < 0.0001). - Ascorbic acid (200 mg/kg) reduced colistin total body clearance.
Yousef et al., 2011 [109]	Melatonin	- The addition of melatonin was associated with lower urinary NAG excretion from day 1 (*p* < 0.0001). - Significant histological abnormalities (*p* < 0.0001) were detected only in the kidneys of the colistin group. Melatonin altered colistin pharmacokinetics, reducing total body clearance.
Ozyilmaz et al., 2011 [106]	*N*-acetylcysteine (NAC)	- NAC addition did not change biochemical parameters but reduced the renal tissue superoxide dismutase level, showing a reduction in oxidative stress parameters.
**Clinical Studies**		
Dalfino et al., 2015 [112]	Ascorbic acid	- 70 patients included. Independent predictors of acute kidney injury (AKI) were baseline renal impairment (adjusted Hazar Ratio, 4.15; 95% CI 1.9–9.2; *p* < 0.001) and age (aHR1.03; 95%CI 1.0–1.05; *p* = 0.028), whereas ascorbic acid was a protective factor (aHR0.27; 95%CI 12–0.57; *p* < 0.001).
Sirijatuphat et al., 2015 [113]	Ascorbic acid	- Nephrotoxicity incidence was 53.8% (7/13) and 60.0% (9/15) in the colistin-ascorbic acid group and the colistin group, respectively (*p* = 0.956). - Urinary excretion rates of neutrophil gelatinase-associated lipocalin and NAG increased during colistin treatment compared to baselines in both groups (*p* < 0.05).
